# Indoor environmental quality assessment of naturally-ventilated school classrooms within a dense arid urban setting of Cairo, Egypt

**DOI:** 10.1038/s41598-025-98602-y

**Published:** 2025-05-09

**Authors:** Samar Afifi, Tarek Kamel, Sherif Ezzeldin

**Affiliations:** 1 Department of Architecture Engineering and Environmental Design, College of Engineering and Technology, Arab Academy for Science, Technology and Maritime Transport –Heliopolis, Cairo, Egypt; 2 Department of Architectural Engineering and Environmental Design, College of Engineering and Technology, Arab Academy for Science, Technology and Maritime Transport – South Valley Branch, Aswan, Egypt

**Keywords:** IEQ, Elementary schools, Students’ satisfaction, Densely populated city, Hot arid climate, Health occupations, Health policy

## Abstract

Indoor Environmental Quality (IEQ) has a significant impact on occupant satisfaction in educational buildings. Key influencing factors include indoor air quality, thermal comfort, visual comfort, and acoustical comfort. These parameters are critical as they directly impact students’ health, academic performance, and learning processes, underscoring the urgent need for research in this underexplored context. The novelty of this study is evaluating the IEQ in elementary school classrooms in Nasr City, Egypt, a densely populated city with a hot, arid climate, while also assessing the differences in IEQ among governmental, private, and experimental schools. This study examines the perceptions of fifth-grade students regarding indoor environmental quality (IEQ) in naturally ventilated elementary school classrooms in Nasr City, Egypt, a densely populated city with an arid climate. The research took a questionnaire approach, and questionnaires were distributed to students across five schools. The responses were then analyzed using SPSS software. The results indicate that generally favorable opinions regarding seating location, natural ventilation, and lighting quality. However, assessments vary by school and classroom; for instance, students seated near open windows reported increased energy levels and focus. This implies that there is a need for strategies to optimize in-classroom layout and ventilation systems, which play a significant role in keeping students engaged in learning activities. However, several challenges were identified, including seasonal discomfort related to temperature fluctuations, excessive noise from street and playground activities, and occasional dissatisfaction with air freshness, especially in classrooms with closed windows. The limitations of this study include a restricted sample size, constraints imposed by local authorities on data collection, and a single-grade focus, which limits the generalizability of the findings. This illustrates the importance of conducting further research to authenticate the results across various educational settings. Ultimately, this study emphasizes the importance of targeted indoor environmental quality (IEQ) improvements in schools to enhance students’ learning experiences and overall well-being. The findings provide valuable insights for policymakers and educational stakeholders by highlighting the main challenges and potential benefits of naturally ventilated classrooms in densely populated, arid urban settings, thereby helping fill a gap in publicly available information concerning IEQ in developing nations.

## Introduction

In educational facilities, Indoor Environment Quality (IEQ) is essential for maintaining teachers’ and students’ performance, well-being, and health. Given that the children spend a significant amount of time in classrooms throughout their school years, the importance of high IEQ in educational settings cannot be overstated^1–6^. Recent reviews have emphasized the critical role of IEQ in influencing educational outcomes, indicating that students thrive in environments that prioritize these factors^3,4^. IEQ includes several factors in a building, such as visual comfort, acoustic comfort, indoor air quality, and thermal comfort^7^. Research has increasingly shown that these factors significantly influence academic performance and overall student well-being, yet there is a notable lack of focused studies within the Egyptian context.

Natural ventilation in classrooms can positively impact students’ performance and general well-being by improving air quality, regulating appropriate temperature levels, encouraging a sense of connection with nature, and encouraging daylighting. However, the efficacy of natural ventilation can be compromised in countries with extreme weather conditions, high noise levels, or contaminated surroundings.

This paper focuses on student satisfaction and perceptions in naturally ventilated classrooms in different school types in the densely populated urban context of Nasr City, Cairo, Egypt. By evaluating Indoor Environmental Quality (IEQ) in this specific setting, the study aims to illuminate the main challenges and potential benefits of naturally ventilated classrooms in dense, arid urban environments. Specifically, the research seeks to answer the following objectives:

1. Analyze the levels of comfort in terms of thermal comfort, indoor air quality, visual comfort, and acoustic comfort in naturally ventilated classrooms.
2. Evaluate students’ perceptions of their learning environment and their level of satisfaction with IEQ factors.
3. Examine the relationship that exists between student performance in different school settings and IEQ factors.

In addition, the results will guide policymakers in the importance of the role environmental issues play in students’ performance, enabling them to take proper steps to enhance students’ learning experiences and achieve significantly better educational outcomes.

This study aims to address the existing research gap regarding the impact of indoor environmental quality in schools in developing countries, with a particular focus on densely populated arid urban settings.

## Literature review

Researchers are increasingly focusing on Indoor Environmental Quality IEQ to determine the optimal conditions for indoor environments. This focus is particularly significant for school classrooms, where children spend extensive time, attracting considerable interest in the literature over the last decade^4–6,8,9^. The necessity of addressing this issue is still increasing, as children are more vulnerable than adults to pollution-related health issues^5,10^. Maintaining healthy IEQ levels is crucial for children’s academic performance and general well-being, especially given that they spend roughly 30% of their day in the classroom under varying environmental conditions^8^.

### Impact of indoor air quality

A key aspect of IEQ is Indoor air quality (IAQ), which has immediate and long-term effects on the occupants’ health^8^. According to ASHRAE standards, at least 80% of occupants should feel comfortable, and there should be no extremely high levels of contaminants in the air or excessive CO_2_ levels^11^. The absence of proper IEQ has been associated with higher absenteeism rates, lower teaching effectiveness, decreased academic achievement, reduced productivity, and a range of health problems, including asthma, throat irritation, shortness of breath, and heart disease across various international studies^12–16^.

Proper ventilation is vital for transforming contaminated indoor air with fresh, clean outside air, particularly in densely populated urban areas^17,18^. Previous studies have shown that lower classroom ventilation rates are associated with poor student attention and increased instances of health complaints^10^. Those who typically inhale air closer to the lower levels of a room are particularly affected by poor indoor air quality, especially in crowded classrooms with low relative humidity^19^.

This supports the results found by Alonso et al., who analyzed pupils’ views regarding thermal comfort and indoor air quality in secondary schools and argued for the positive effect of sufficient IAQ on students’ comfort and learning achievement in various educational contexts^20^. This is further supported by a study conducted by Amoatey et al. on high school buildings in the region during the moderate winter season, which correlated students’ views on the indoor environment with their academic performance and health in a comparable climate^21^.

### Impact of thermal comfort

Thermal comfort is a frequently researched factor that impacts IEQ in schools, particularly elementary schools located in hot, arid regions like Egypt^22^. Defined as the mental state of satisfaction with the thermal conditions within a building, thermal comfort is influenced by several variables, including age, gender, location, season, and individual adaptability among various student populations^19,23^. Maintaining thermal comfort is vital to enhancing students’ focus and productivity in naturally ventilated classrooms. Specifically, temperature, humidity, and ventilation need to be adjusted to meet the students’ and educational needs^24^.

Several studies highlighted that student performance significantly decreases when temperatures exceed 26 °C, with mathematical ability being highly sensitive to temperature fluctuations as confirmed by other international research^19,25^. For example, Cătălina and Banu observed that children performed 16% worse in classrooms with elevated temperatures compared to those in optimal conditions and 12.63% worse in classrooms excessively cold temperatures^26^.

Andersen and Gyntelberg suggest the indoor air temperatures should not be less than 24 °C to ensure thermal comfort for 85% of the occupants in different settings^27^. Some studies suggest that students perform best when indoor air temperatures are maintained between 26 and 29 °C which maximizes engagement and productivity^23^. Conversely, Soltaninejad noted that excessively high temperatures can decrease performance and increase student discomfort and potential health risks^15^.

Recent research has expanded on these findings. For instance, Romero et al. examined thermal comfort as an influencing factor on the academic productivity of university students, noting that averting temperature extremes can significantly increase student participation and concentration in higher education contexts^28^. This supports our discussion on thermal conditions in elementary and secondary schooling. Moreover, Lin and Zhang looked into the interactions of stress, depression, and emotion with thermal comfort and, once more, emphasized the importance of sufficient thermal comfort in classrooms for the better health of the students in both elementary and secondary education^29^.

Recent insights of Alonso et al. elaborate further on the correlation between thermal comfort and student perception. For example, their results reveal human comfort is significantly reduced, for example, 21% of students reported being ‘very uncomfortable’ at temperatures greater than 34 °C. Hence, 40% students feel ‘comfortable’ at 27 °C, but discomfort levels above this temperature are unreasonably high. The study also found that 60% of occupants are comfortable at 27 °C during the non-heating season, but 40\% discomfort is experienced at 19 °C during the heating season. Such data clearly portrays the fact of how constantly changing student thermal comfort is and it emphasizes the critical need of setting proper temperature limits in classrooms to promote effective learning environments^20^.

### Impact of visual comfort

Natural lighting and ventilation are essential components in designing effective educational environments for elementary school classrooms. Previous research highlights several benefits for teachers and students when classrooms are well-lit with natural light. Students who attend classes with ample natural light tend to perform better academically, often scoring higher in reading and math than their peers in inadequately lit environments, according to a study conducted by the Heschong Mahone Group^30^. Furthermore, adequate natural light is associated with improved visual comfort, reduced eye strain, and enhanced teacher and student productivity across various learning environments^19^.

By optimizing classroom design to maximize natural light and ventilation, teachers can foster learning environments that support academic success while also reducing carbon emissions and school operational costs^15,31^. Classrooms with appropriate lighting and ventilation enhance comfort and promote increased engagement, productivity, and focus among students and teachers, as evidenced in various educational settings^32,33^. Additionally, exposure to natural light assists in regulating circadian rhythm, subsequently improving sleep quality and overall well-being^24,27^. Thus, schools can significantly enhance students’ physical health and holistic development by prioritizing the integration of natural light into classroom designs as part of IEQ optimization efforts^34^.

Recent research by Lin and Zhang observed how different mental states influence the perception of light. Stressed participants only found the lighting suitable 30% of the time, and an astonishing 79% of those with negative emotions considered the lighting either too bright or too dark. On the other hand, unstressed participants were neutral about the lighting. This emphasizes the need to provide appropriate lighting for different psychological conditions, mostly in schools where students undergo varying degrees of stress and emotional challenges^29^.

### Impact of acoustic comfort

In educational settings, noise is a serious public health concern that adversely affects children’s growth and well-being^35–37^. Challenges such as noise disruption, thermal discomfort, glare, and inadequate ventilation significantly hinder learning environments and reduce educational outcomes ^12,34^. Of particular concern is the detrimental effect of noise on students’ academic performance and overall classroom experience^38,39^. Exposureto high noise levels can lead to increased fatigue and potential hearing issues among students^17,40^.

De la Hoz-Torres and Kapoor emphasize that excessive noise diminishes the overall quality of the teaching and learning process by disrupting acoustic clarity^19,41^. Poor acoustics are linked to heightened stress, sleep disturbances, diminished attention spans, and reduced levels of student comfort, productivity, and overall well-being^19^.

The growing research on indoor environmental quality (IEQ) in classrooms underscores the impact of various environmental factors on student health and academic performance. Recent studies highlight the critical importance of Indoor Air Quality (IAQ) management systems. For instance, a study by Haverinen-Shaughnessy found that schools with regular maintenance and effective ventilation strategies reported lower rates of respiratory issues and improved student attendance^9^. Additionally, research by Wargocki and Wyon demonstrated that enhanced ventilation rates not only boost cognitive function but also reduce perceived discomfort, thereby fostering a more conducive learning environment^42^.

These findings clearly link well-managed indoor environmental quality factors, comprising visual, acoustic, IAQ, and thermal comfort. Given the unique climatic challenges in cities like Nasr City Cairo, ongoing research is essential to identify solutions that address specific environmental needs, ensuring that educational institutions promote health and effective learning.

## Research gap

Earlier studies have investigated specific IEQ factors including indoor air quality, thermal comfort, visual comfort, and acoustic comfort. However, there is a knowledge gap about the impact of these factors on elementary school student’s academic performance and well-being in hot arid regions and densely populated cities. Additionally, many available studies try to analyze the problems in different climatic and density locations, creating a gap in evaluating these factors in Cairo, Egypt, and other similar contexts within different classroom settings. Thus, this study aims to investigate the combined effects of various IEQ elements on students’ performance in these rigorous environments, thereby advancing the understanding of educational practices and policies tailored to these settings.

### Novelty of the work

The goal of this study is to evaluate the Indoor Environmental Quality (IEQ) in elementary school classrooms in Nasr City, Egypt, a densely populated city located in a hot, arid region. It also aims to assess the differences in IEQ among different types of schools: governmental, private, and experimental, and to offer strategies to alter these conditions. Furthermore, this study incorporates the capturing of elementary school students’ perspectives on such IEQ issues, enhancing their insights and understanding towards the learning environment.

The objective of this research is to assess the Indoor Environmental Quality in the naturally ventilated classrooms in Cairo included the following: Obtaining information about students’ evaluation of indoor air quality about freshness, odors, and comfort in naturally ventilated classrooms, as well as evaluating whether or not natural ventilation helps create a suitable learning atmosphere. Obtain data related to students’ levels of comfort regarding classroom temperature in different seasons and analyze the most frequently mentioned reasons for thermal discomfort. Find out the level of noise in the classroom that is distracting, the communication and concentration levels during the lessons, and the elementary causes of the noise disturbances as identified by the students.

## Methodology

This study targets elementary schools in Nasr City, Cairo, Egypt, which represents a typical dense urban area of Cairo following an orthogonal planning grid of repetitive building blocks having the same orientation. By focusing on the 6–12 age group, this research acknowledges the increased vulnerability of children to health problems compared to adults, and a significant percentage of children in this age group are at the elementary stage which is suggested in previous research^5,10^, as well as the significant dropout rates at the elementary education level due to financial challenges.

**Fig. 1:**
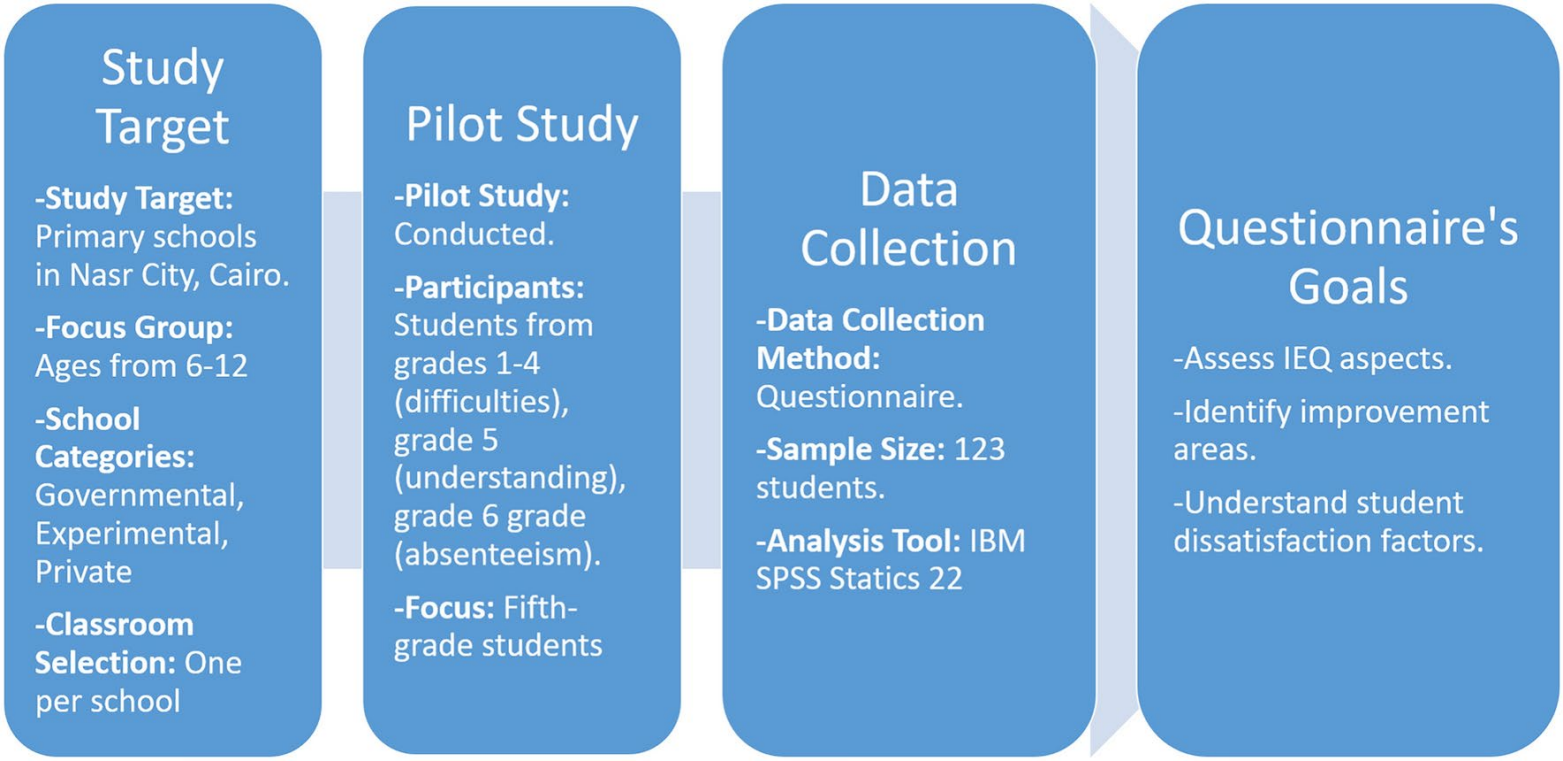
Research methodology diagram.

The study will examine the three Egyptian elementary school categories: governmental, experimental, and private. Five classrooms, one classroom per school from five different schools, will be evaluated across these school types, delivering a thorough examination of the different educational settings in this urban context. Each type of school comes with different student density and academic approaches, which adds more value regarding the understanding of IEQ. The selection of the five classrooms, each representing a representative classroom from each of the five different schools, captures a diverse range of students in the educational environment of Nasr City. Figure 1 illustrates the research methodology diagram, which outlines the complete methodology of this research. It illustrates the flowchart of steps taken to gather students’ perceptions on Indoor Environmental Quality (IEQ) in naturally ventilated classrooms.

Before the main study, a pilot study was done in one school to test how well the students of different elementary school grade levels understood the questionnaire. According to the results, students from grades one through four had difficulties reading and responding to the questions, while fifth graders understood the questionnaire and provided thoughtful answers. Consequently, fifth-grade students have been chosen as the primary focus for data collection in each selected classroom prototype. The study did not include sixth-grade students because of their frequent absenteeism, as there were no grades for attending classes in school. This targeted approach allows for a focused examination of the fifth-grade students.

This study adopts a mixed-methods approach, combining both quantitative and qualitative elements. A quantitative objective approach utilized questionnaires; however, due to privacy and security concerns, experimental measures could not be conducted in classrooms. This methodology allows for a holistic approach to assess all IEQ aspects, prioritizing critical parameters to be evaluated later in future studies. The study selected five schools with permission from the Egyptian Ministry of Education. The comparable urban layout and density of the surrounding residential buildings of these schools ensure consistency in the study’s environmental context. The use of IBM SPSS Statistics 22 for data analysis after 123 students answered the study’s questionnaire further strengthens the reliability of the findings.

The main goal of this questionnaire is to collect data and assess various aspects of IEQ, pinpoint areas that require improvement, and determine the factors that dissatisfy the students. By assessing students’ perceptions and experiences with IEQ factors, the questionnaire intends to provide insights that can be used to guide initiatives and policies that promote more comfortable and healthy indoor settings.

The questionnaire aims to provide a comprehensive assessment of students’ well-being and satisfaction throughout the school year by focusing on key months of the academic year (October, January, and April). These months correspond to distinct seasonal conditions that are critical to understanding variations in IEQ. By collecting data from certain months based on students’ prior experiences, the questionnaire aims to provide an in-depth understanding of how environmental conditions patterns impact student well-being throughout the school year.

### Selected case study description

The questionnaire was conducted on five classroom prototypes in different educational institutions. The classrooms in Schools A, B, and E were orientated northwest, whereas those in School C were orientated northeast, and in School D were oriented towards the East. Table 1 gives a summary configuration analysis of the five listed classrooms, which illustrate different types of schools in Nasr City in Cairo, Egypt. The descriptions analyze fundamental features, including the area, orientation, classroom context, dimensions, student density, and ventilation methods. The classification of the schools into types appeared in the table (private, experimental, and government) provide more comparison of the diverse schools settings. All selected classrooms are on the upper levels to mitigate the noise from the playground, ensuring that the study controls for external disturbances. The selected classrooms in Schools A, B, C, and E are on the fourth floor, whereas the chosen classroom in School D is on the third floor as this school has only three floors. Classroom capacities range from 18 to 60 students, with two windows on the exterior wall and one facing the corridor. The questionnaires were distributed to students in November 2023 during mild temperatures. According to the Egyptian Meteorological Weather Station, temperatures in Nasr City ranged from 21 to 26 °C, with wind speeds ranging from 10 to 12 km/h.

**Table 1:**
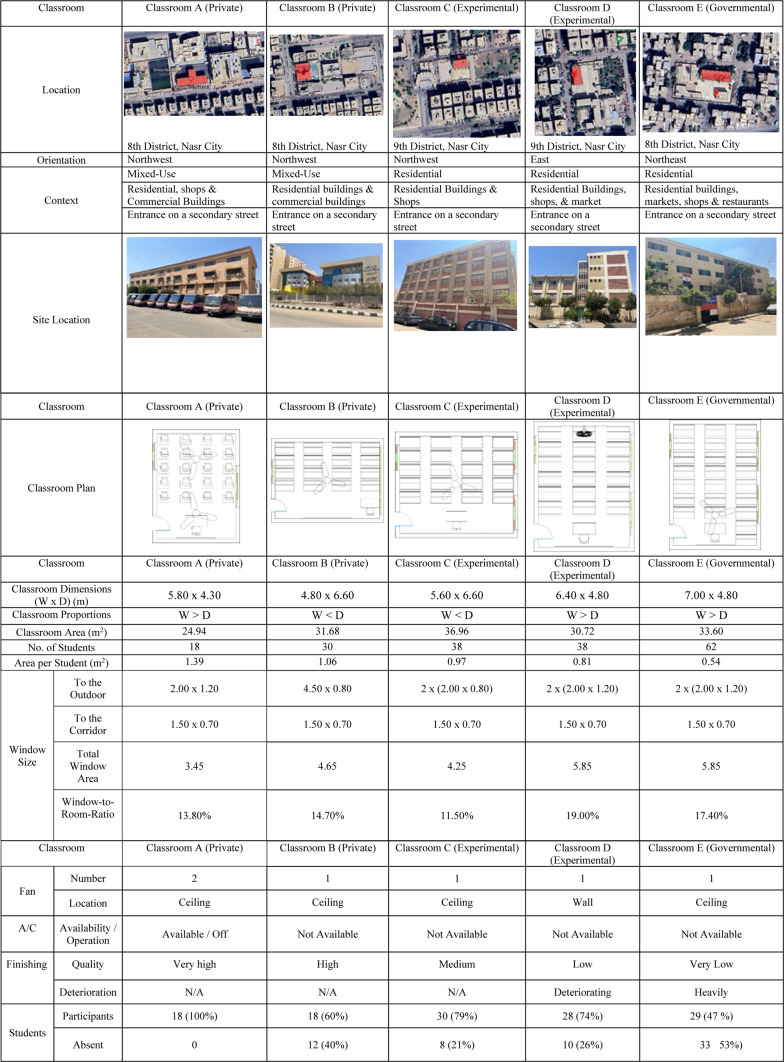
Detailed Description for the five chosen selected classrooms each in different school covering the three types of schools in Nasr City, Cairo Egypt.

POST-FLIGHT: individual touchdown parameters for the four post-flight landing simulations, two on the day of return (R + 0–1) and one each on R + 3–4 and R + 7–9. Post-flight group mean is also shown.

Through the analysis each school was examined independently, and then all students’ responses were compared. The questionnaire included nominal and ordinal variables to capture a variety of student replies adequately.

### Questionnaire design

The survey, which employed a 5-point Likert Scale with multiple-choice items^6,43,44^, was administered to students in both Arabic and English to assess their level of satisfaction during morning courses in November 2023. The study employed a 5-point Likert scale in the questionnaire. This scale is frequently used in survey research, enabling students to indicate their feelings using a symmetric scale. This scale measures the relative importance of respondents’ attitudes toward each question, which spans a symmetric agree-disagree range, producing a pattern with scaled characteristics^45,46^.

**The questionnaire was divided into six sections:**

- Student Information: This section collects nominal data, including age, school year, classroom location, and seating arrangement.
- Seating Satisfaction: In October, January, and April, students evaluated their satisfaction with their seats and the state of the classroom windows during both class time and break periods in the classroom. Based on the students’ prior experiences, this section used ordinal variables with the Likert scale.
- Visual Comfort: This section evaluated students’ satisfaction with the board and desk illumination, the proximity of the windows, and any outside-view distractions.
- Acoustic Comfort: Students evaluated how well they could hear their teacher and identified areas where the noise disrupted the class.
- Indoor Air Quality: Students discussed the types of pollutants that affect them, the frequency of dust detection, the availability of fresh air, and the classroom’s odor.
- Thermal Comfort: This part evaluated students’ comfort levels in both morning and afternoon sessions in October, January, and April. To measure the clo value, which represents clothing insulation, students chose pictures representing the types of clothes they wore during the questionnaire.

Students also ranked the four parameters of IEQ—thermal Comfort, Air Quality, Acoustic Comfort, and Visual Comfort—according to their importance.

The Central Agency for Public Mobilization and Statistics, the Egyptian Ministry of Education, the Cairo Education Directorate, and the East Nasr City Educational Administration, which oversees the five schools involved in the study, approved the distribution of the questionnaire to the selected schools.

### Descriptive data analysis

IBM SPSS Statistics (version 22) was used to analyze the questionnaire data through descriptive statistical analysis. To identify correlations within the data, Pearson correlation tests, mean satisfaction scores (MSS), and percentages were also conducted.

## Results

### Overview of indoor air quality

According to the questionnaire results, students’ opinions regarding their energetic feelings in the classroom are relatively evenly distributed. Part of them indicate that they feel energized, while the other part report feeling sleepy. The overall mean satisfaction score for students’ perceptions of their energy levels is 3.10 out of 5 (σ = 1.51). Further analysis reveals that students seated at the back in the classroom, particularly those away from the windows, are more likely to feel sleepy, while students seated at the front, nearer the window report feeling more energized. With an overall mean score of 3.28 out of 5 (σ = 1.43), most students are satisfied with the fresh air in the classroom. Notably, most girls often sit closer to windows and express satisfaction with the fresh air. In contrast, most boys set farther from the windows show dissatisfaction with the amount of fresh air available.

Despite some satisfaction with fresh air, many students express dissatisfaction with the classroom smell, reflected in a mean satisfaction score of 2.33 out of 5 (σ = 1.29), which is below the neutral Likert scale value of 3 as shown in Table 2. This table presents the mean satisfaction scores and standard deviations (SD) regarding various aspects of indoor environmental quality as reported by students. This results indicates significant discomfort with indoor odors, suggesting ventilation systems is insufficiently addressing smell issues both inside and outside the classroom.

**Table 2:** Student responses on satisfaction and comfort levels.

Students response	Mean satisfaction	SD
Students feeling energized	3.10	1.51
Students are satisfied with the amount of fresh air	3.28	1.43
Students are unsatisfied with the classroom smell	2.33	1.29

Additionally, 62% of students reported that they “sometimes” notice dust in the air, which could be related to the schools’ location in Nasr City, Cairo, characterized by frequent sandy weather. Only 43% of students indicated they are not disturbed by any particular sources of air pollution in the classroom, however, 35% reported disturbance from outdoor air pollution sources, while 22% cited indoor air pollution as a source of irritation, as shown in Table 3. This table outlines students’ mean score and standard deviation (SD) for satisfaction with several features of indoor environmental quality.

**Table 3:** Student responses on air quality concerns.

Question	Majority of students’ response	%
Dust in classroom	Sometimes	62
Air pollution sources	None	43
	Outdoor air pollution sources	35
	Indoor air pollution sources	22

This data indicates a concerning perception of air quality among the students, which implies that there is a need for effective air quality management.

### Overview of thermal comfort

The author distributed the questionnaire physically in November 2023. It received 123 responses; Table 4 demonstrates the number of students who responded to the questionnaire in each classroom type, and the percentage of boys and girls who participated in the questionnaire in each classroom.

**Table 4:** The number of students who responded to the questionnaire.

Classroom Type	N (Boys)	% (Boys)	N (Girls)	% (Girls)	Total N
Classroom A	9	50	9	50	18
Classroom B	11	61	7	39	18
Classroom C	16	53	14	47	30
Classroom D	13	46	15	54	28
Classroom E	9	31	20	69	29
Total	58	47	65	53	123

Table 5 presents the thermal insulation values (clo) measured for students during various class times throughout the educational year. The majority of students felt cold during the November morning classes, prompting them to wear long-sleeved jackets and trousers, resulting in a clothing insulation (clo) value of 0.91. After physical activities during break time, they remove the jacket, which changes the clo value to 0.55. In April, most students felt warm throughout the school day and wore short-sleeved T-shirts and trousers, resulting in a clothing insulation (clo) value of 0.49. In January, most students felt cold in morning classes and neutral in afternoon classes, wearing long-sleeved T-shirts and trousers, which resulted in a clothing insulation (clo) value of 0.55. Such measurements reveal the differences in students’ thermal comfort levels, which help identify how changes in seasons affect the indoor environmental quality of the classrooms.

**Table 5:** clo. Throughout the educational year.

Time of classes	clo
November morning classes	0.91
November afternoon classes	0.55
April, through the day	0.49
January through the day	0.55

Most students stated that in October, the windows were opened throughout the day while the fans were turned off. In January, both the windows and the fans remained closed during the school day. In April, most students reported opening windows and turning fans on during the school day, suggesting seasonal adaptations to thermal comfort conditions.

Table 6 illustrates the students’ perception of thermal comfort at different times during the academic calendar year. It reveals information regarding most student satisfaction during different class periods and their feedback of comfort related to the classroom environment.

**Table 6:** Students feeling during the school day throughout the school year.

Time of classes	Majority of satisfaction
October morning classes	Neutral
October afternoon classes	Warm
January morning classes	Very cold
January afternoon classes	Neutral
April morning classes	Neutral
April afternoon classes	Neutral

In terms of ventilation, the majority of students reported general satisfaction with an acceptability mean score of 3.59 out of 5 (σ = 1.31), indicating a perception of adequate ventilation in their classrooms, which is significantly above the neutral value of 3 on the Likert scale.

### Seating preferences and satisfaction

An analysis of seating preferences revealed that most boys preferred seating positions farther from the windows and the teaching area. In contrast, most girls preferred seating nearer to the windows and the teaching area. Using a Likert 5-point Likert scale, students expressed their satisfaction with their seating locations, achieving an average score of 4.35 out of 5 (σ = 0.94), with 59.3% of students expressing high satisfaction.

Students also reported that windows were kept open all day in October, periodically opened in January, and consistently open all day in April. Their satisfaction with proximity to the window yielded a mean satisfaction score of 4.09 out of 5 (σ = 0.87), indicating a general satisfaction with seating locations in terms of window placement.

Statistical analysis indicated no significant difference in responses between boys and girls regarding satisfaction with seating location or perceptions of indoor air quality.

### Overview of visual comfort

Regardless of gender, most students are generally satisfied with the lighting quality on the classroom board. Table 7 presents the mean satisfaction scores and standard deviations (SD) for students’ responses to visual comfort regarding classroom lighting and outdoor views. The data shows that the overall satisfaction mean score for the lighting quality on the board is 3.30 out of 5 (σ = 1.22), which exceeds the neutral Likert scale value of 3, indicating a general level of satisfaction among students. Furthermore, students expressed even greater satisfaction with the lighting quality at their desks, achieving a higher mean score of 4.35 out of 5 (σ = 1.04). This indicates that 63% of students are satisfied with the lighting quality on their desks, highlighting favorable opinions about lighting conditions in the classroom. In contrast. 40% of students reported a mean score of 2.93 out of 5 (σ = 1.24) regarding the outdoor view distraction level, which is closer to the neutral value of 3.

**Table 7:** Visual comfort satisfaction questions.

Questions	Mean satisfaction	SD
Lighting quality on the board	3.30	1.22
Lighting quality on the desks	4.35	1.04
Outdoor view distraction	2.93	1.24

This suggests that most students are not significantly distracted by the outdoor views, potentially related to the residential surroundings of the chosen schools. Both boys and girls reported similar levels of distraction concerning the outdoor views outside their classroom, indicating a consistent perception across genders.

### Overview of acoustic comfort

Approximately 60% of students feel inconvenienced by street noise, which includes disturbances from the playground, the corridor beside the classroom, and even noise within the classroom itself. Boys indicated that noise from the corridor is their primary source of disturbance, while girls report that noise within the classroom is more disruptive to them.

Even with these noise disruptions, general satisfaction with being able to hear the teacher clearly remains high at score of 4.34 out of 5 (σ = 0.97) which is above the neutral score of 3. In detail, 61% of students declare that they are highly satisfied with hearing the teacher as a result of teacher’s sound level, which often exceeds background noise. Some students, however, note that the sound level discomforts them and has a negative effect on their overall learning experience.

### Correlations

The results show that students’ satisfaction with fresh air and outdoor views is significantly correlated with their gender (*p*-value = 0.001), indicating distinct perspectives among boys and girls on these aspects inside the classroom. Furthermore, a correlation exists between seating location—specifically, proximity to the nearest window—and multiple factors related to indoor environmental quality (IEQ). These factors include lighting on the desk (*p*-value = 0.007), teacher hearing satisfaction (*p*-value = 0.001), fresh air satisfaction (*p*-value = 0.001), and airflow in classroom satisfaction (*p*-value = 0.000). This analysis implies that during classes, students who are seated next to open windows might have some level of glare on their desks which can distract them from following the lesson appropriately.

Simultaneously, these students tend to be more satisfied with the amount of fresh air and the airflow in the classroom. Thus, seating location—whether near or far from windows—has a significant impact on various IEQ factors.

Additionally, there is a significant correlation between the state of windows (open or closed) throughout the school year and students’ satisfaction with the smell of the classroom with p-values ranging from (0.000 to 0.008). This finding suggests that window operation significantly impacts air quality and ventilation efficiency, subsequently influencing students’ perceptions of the classroom smell.

Table 8 summarizes these correlations in order to highlight how strategically managing windows for ventilation and optimizing seating arrangements enhances student comfort and satisfaction in the classroom.

**Table 8:** Correlations found from the questionnaire between indoor environmental quality parameters.

Parameter	Sub-parameter	Mean	Std. Deviation	Correlation with gender		Correlation with seat location satisfaction		Correlation with window proximity	
				r value*	*p*-value**	r value*	*p*-value**	r value*	*p*-value**
Visual comfort	Outdoor view satisfaction	2.930	1.249	− 0.291	0.001**	− 0.165	0.068	− 0.046	0.611
	Lighting quality on the desk	4.350	1.048	0.051	0.574	− 0.041	0.649	0.242	0.007**
Air quality	Fresh air satisfaction	3.280	1.439	0.307*	0.001**	0.247	0.006**	0.285	0.001**
Thermal comfort	Students’ thermal comfort during the questionnaire	3.020	0.689	− 0.025	0.783	− 0.247	0.006**	− 0.030	0.746
	Airflow in Classroom Satisfaction	3.590	1.318	0.086	0.343	0.261	0.004**	0.351*	0.000**
Acoustic comfort	Teacher hearing satisfaction	4.340	0.974	-0.037	0.686	0.233	0.009**	0.290	0.001**

*Correlation coefficient (r < 0.3 weak, r = 0.3–0.5 medium, r > 0.5 strong).

**Correlation is significant at *p* < 0.05.

### Grouping analysis

When the five classrooms are grouped by school type, students’ opinions on IEQ demonstrate three distinct patterns across different school types:

Private schools (Group 1), experimental schools (Group 2), and governmental schools (Group 3).

- Group 1 (Private Schools):
  - 43% of students express satisfaction with the amount of fresh air inside their classroom, while 30% report neutrality, and 27% indicate dissatisfaction.
  - 76% of students express dissatisfaction with the classroom smell.
  - 63% of students report that no perceived air pollution resources affect them.
  - 44% of students report noise disruptions from the corridor activities and classroom noise, 40% from the playground sounds, and 16% from street noise.
  - 82% of students do not find outdoor views distracting.
- Group 2 (Experimental Schools):
  - 44% of students report dissatisfaction with the fresh air in their classroom; however, 32% feels satisfied, and 24% remain neutral.
  - 51% of students express dissatisfaction with the classroom smell, 31% are neutral, and only 18% are satisfied with it.
  - 57% of students report no available air pollution sources affecting them, while 25% state that outdoor air pollution sources are bothersome, and 18% acknowledge indoor air pollution sources.
  - 43% of students are disturbed by noise from streets and corridors, 33% are disturbed by noise from the playground, and 24% are disturbed by noise from inside their classroom.
  - Interestingly, 79% of students perceive outdoor view as not distracting.
- Group 3 (Governmental):
  - 52% of students report that outdoor air pollution sources affect them, while 31% are disturbed by indoor air pollution sources, and 17% state that no available air pollution sources affect them.
  - 56% of students reports dissatisfaction with their classroom smell.
  - Disturbances from street and corridor noise affect 52% of students, while 35% of students are disturbed by playground noise, and 13% cite disturbances from sounds inside their classrooms.
  - 56% of students observed outdoor views as very distracting, likely due to the nearby street markets and surrounding loud noises.

These results indicate that compared to classrooms in private schools, classrooms in both experimental schools and governmental schools generally experience lower levels of satisfaction regarding acoustic comfort, visual comfort, and air quality. The governmental school has reported a high impact of outdoor pollutants and street distractions which demonstrates there is a wide range of environmental differences which have an effect on the comfort and satisfaction of the students.

### Indoor air quality (solely)

**Fig. 2:**
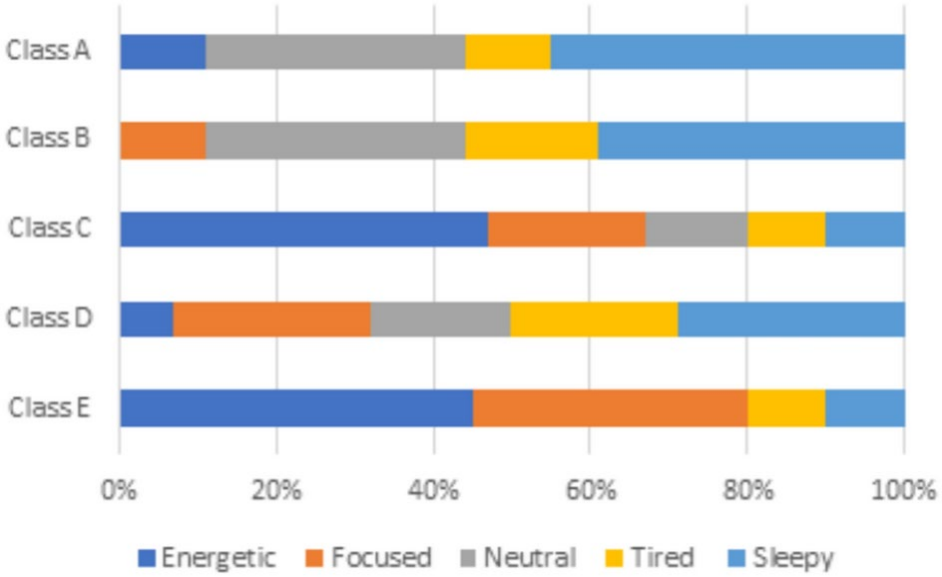
Assessment of students’ emotions within the classroom setting.

**Fig. 3:**
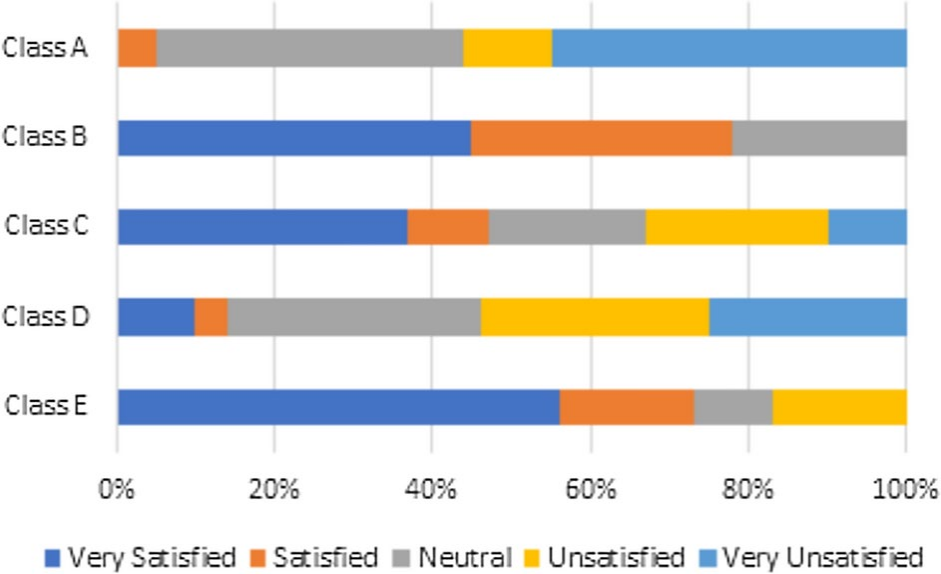
Assessment of students’ satisfaction with the amount of fresh air in their classroom.

**Fig. 4:**
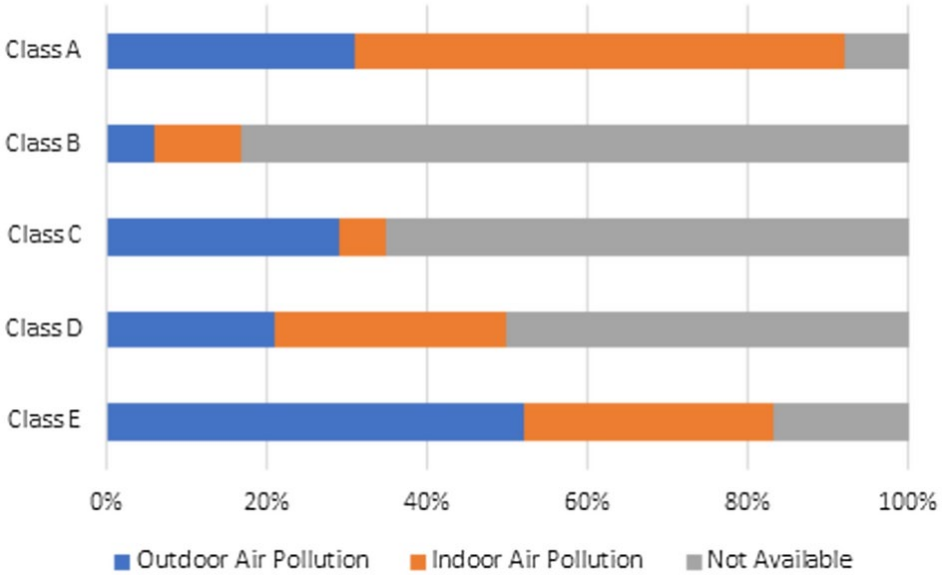
Assessment of air pollution sources in the classroom.

Different patterns appear when analyzing students’ assessments in various classrooms based on environmental factors such as seating arrangements, ventilation methods, and surrounding pollution sources (see Figs. 2, 3 and 4):

- Classroom A:
  - 45% of students report feeling sleepy, and 11% feel tired, potentially due to insufficient fresh air circulation. The only source of ventilation is a single air conditioner, which keeps the windows closed.
  - 56% of students express dissatisfaction with the fresh air quality.
  - 61% of students report observable disturbances from indoor air pollution, while 31% indicate that outdoor air pollution affects them, and 8% report no available air pollution sources causing disturbances.
- Classroom B:
  - 78% of students express satisfaction with the fresh air provided by the open windows, which facilitates natural ventilation.
  - 39% of students report feeling sleepy, and 27% feel tired.
  - 83% of students indicate that no available air pollution sources disturb them. In comparison, 11% of students report that indoor air pollution sources disturb them, and only 6% report that outdoor air pollution disturbs them.
- Classroom C:
  - 47% of students report feeling energetic, and 20% feel focused, particularly among those seated near the windows.
  - 65% of students report no disturbances from air pollution sources, while 29% state that outdoor air pollution affects them, and 6% indicate disturbances from indoor air pollution sources.
  - 47% are satisfied with the amount of fresh air provided by the open windows as they are seated close to the windows; however, 33% express dissatisfaction due to seating farther from the windows, and 20% remain neutral regarding the fresh air supply.
- Classroom D:
  - 29% of students report feeling sleepy, and 21% report feeling tired, possibly linked to increased indoor density.
  - 50% of students report no disturbances from air pollution sources, while 29% cite disturbances from indoor air pollution sources, and 21% report disturbances from outdoor air pollution sources.
  - 54% of students express dissatisfaction with the amount of fresh air provided by the open windows due to their seating position. In comparison, 32% remain neutral, and only 14% are satisfied with the amount of fresh air provided.
- Classroom E:
  - 45% of students feel energized, and 35% feel focused, particularly those sitting near open windows.
  - 52% of students report disturbances from outdoor air pollution sources, while 31% are affected by indoor air pollution sources, and 17% report no disturbances from available air pollution sources. This indicates that outside air pollutants—from local street markets, garbage, restaurants, car emissions, and cigarette smoke—profoundly impact student experience**,** despite the benefits of open-window ventilation.

Research indicates that natural ventilation through windows generally enhances students’ perceptions of energy levels and air quality; however, these benefits can be compromised by proximity to high pollution levels. This highlights the need for protective interventions that mitigate external pollutants and enhance internal air circulation in schools located in busy or densely populated areas. The variety in experiences from different classrooms suggests the need for more proactive approaches in addressing environmental challenges through flexible as well as holistic IEQ strategies aimed at improving the learning environment.

Figure 2 illustrates students’ perception of energy levels in the classroom. Figure 3 demonstrates students’ satisfaction with the amount of fresh air in the classroom. Figure 4 illustrates the analysis of air pollution sources identified by students in various classrooms.

### Thermal comfort (solely)

**Fig. 5:**
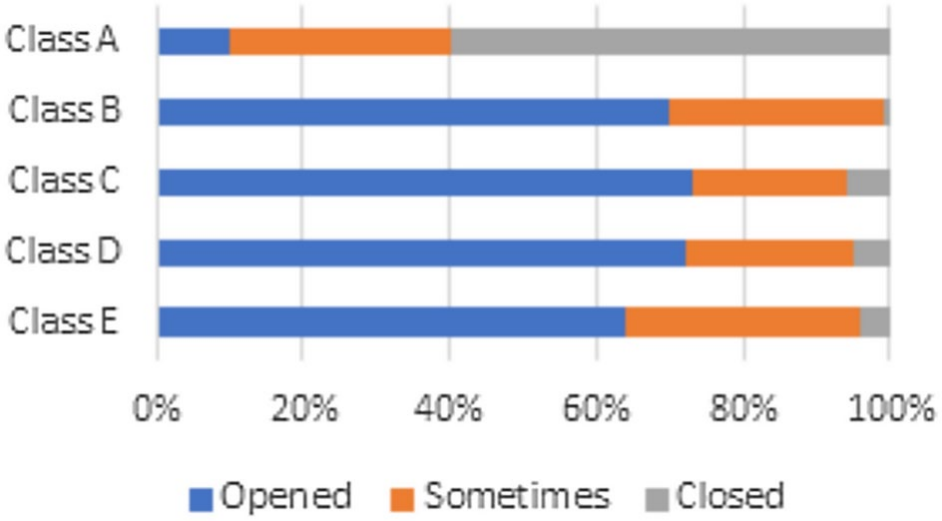
Windows state during school day in october.

**Fig. 6:**
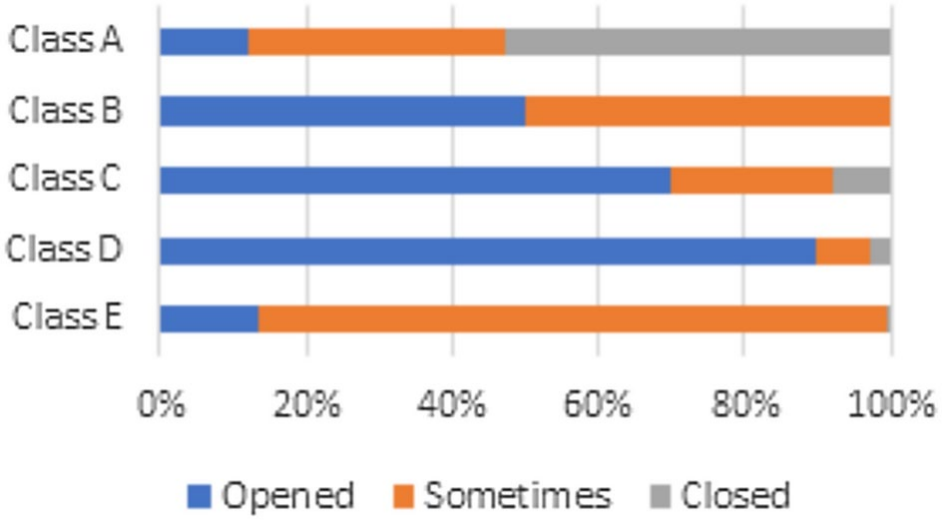
Windows state during school day in April.

Students’ assessments of ventilation and airflow reflect distinct patterns when comparing classroom-specific variances across different types of schools:

- Classroom A: Most students stated in October and April that windows are kept shut throughout the school day because there is only one air conditioning unit available for ventilation. Consequently, many students indicated feelings of insufficient airflow (see Fig. 5). This figure illustrates the state of the classroom windows in October, focusing on the relationship between window state and indoor environmental conditions.
- Classrooms B, C, D, and E: In contrast, students in these classrooms reported that windows are their only source of ventilation, leading to the observation that windows remain open throughout the school day in both October and April. The open-window policy leads to improved levels of satisfaction in terms of thermal comfort (see Fig. 6). This figure illustrates the state of the classroom windows in April. It provides an analysis in comparison to October, highlighting differences in window opening and closing.

#### Visual comfort (solely)

Students’ satisfaction with outdoor views and lighting quality reveals significant differences between classroom types:

- Lighting Quality on Desks:
  - Classroom A: 62% of students report satisfaction with the lighting on their desks.
  - Classroom B: 55% of students express satisfaction with the lighting on their desks.
  - Classroom C: 46% of students report being satisfied with the lighting on their desks.
  - Classroom D: 46% of students report dissatisfaction with the lighting on their desks.
  - Classroom E: 46% of students report satisfaction with the lighting on their desks.

**Fig. 7:**
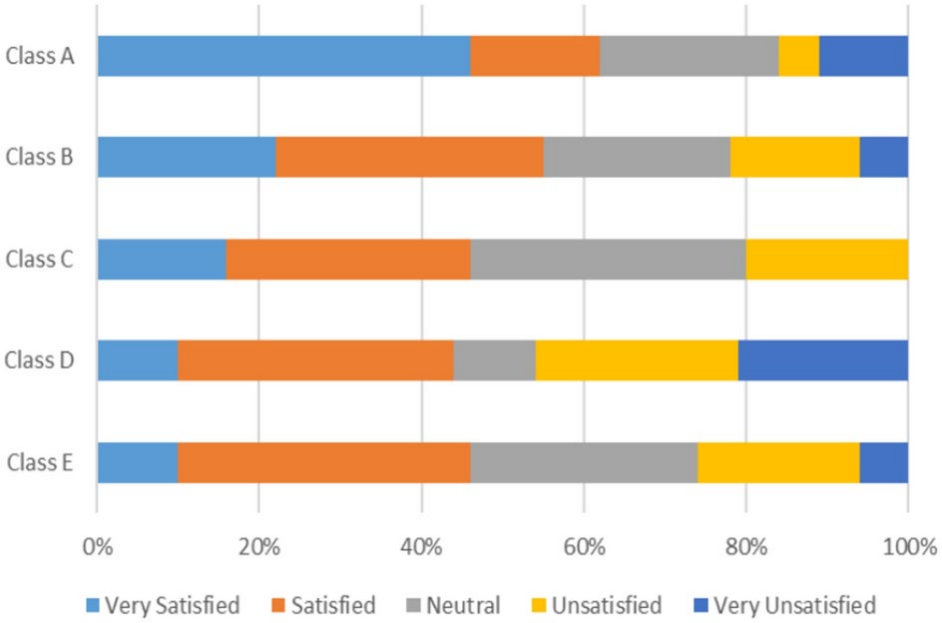
Assessment of students’ satisfaction with the lighting quality on the desks.

These findings indicate that natural and artificial light are generally sufficient for desk-oriented tasks (see Fig. 7). This figure assesses student satisfaction regarding the illumination provided at their desks. It illustrates results concerning students’ evaluations on the degree of light, its constancy during the work, and the level of comfort experienced while seated at the desks.

- Lighting Quality on Boards:
  - Classroom A: 73% of students are satisfied with the lighting on the classroom board.
  - Classroom B: A notable 90% of students express satisfaction with the lighting on the classroom board.
  - Classroom C: 86% of students report satisfaction with the lighting on the classroom board.
  - Classroom D: 87% of students express satisfaction with the lighting on the classroom board.
  - Classroom E: 91% of students are satisfied with the lighting on the classroom board.

**Fig. 8:**
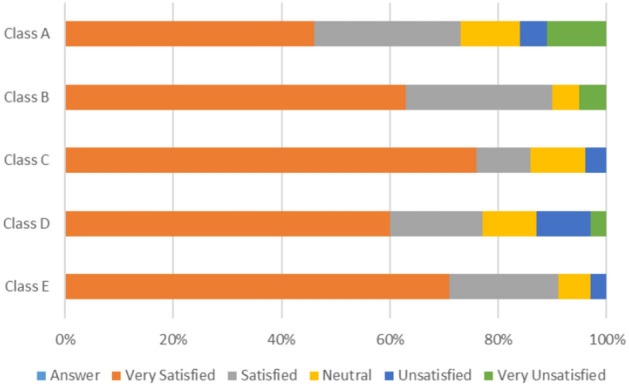
Assessment of student satisfaction with the lighting quality on board.

This demonstrates adequate light levels for visibility during lessons (see Fig. 8). This figure shows the students’ satisfaction with the quality of lighting on the board. The evaluation measures students’ assessment of the visibility and effectiveness of the lighting on the board, which is essential for effective teaching and learning.

**Fig. 9:**
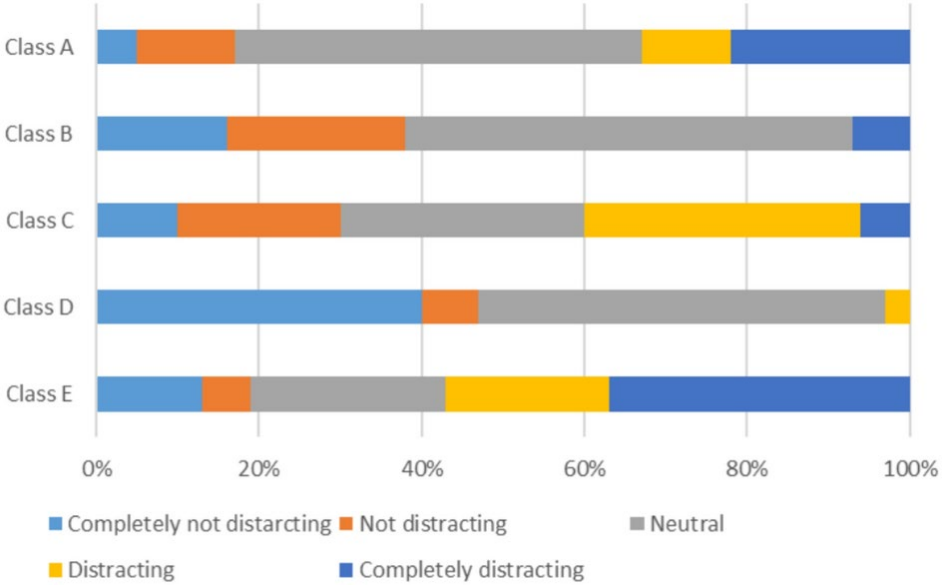
The assessment of outdoor view degree of distraction for students.

**Fig. 10:**
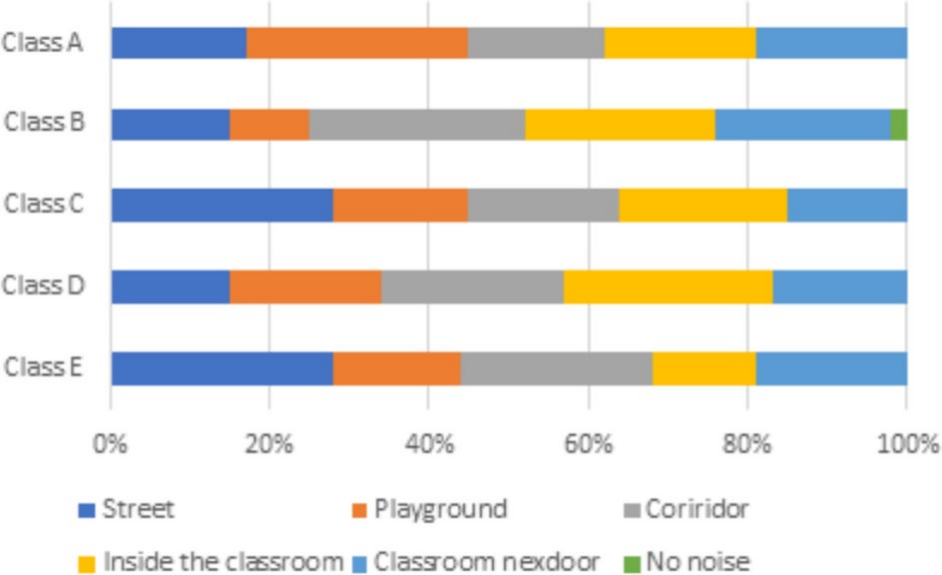
Noise sources.

- Outdoor Views: There are significant variations in how students perceive outdoor views (see Fig. 9): Figure 9 presents an analysis of how distractions from outdoor views impact students during class.
  - Classroom A: 50% of students are neutral regarding outdoor views, suggesting these views in these spaces do not significantly interfere with their concentration. Meanwhile, 33% are distracted, and 17% report that outdoor views do not distract them.
  - Classroom B: 55% maintain a neutral stance, while 38% are not distracted by the outdoor views, indicating minimal impact on concentration. Only 7% find outdoor views distracting.
  - Classroom C: 40% report distraction from the outdoor views due to visually stimulating activities, while 30% are not distracted, and another 30% are neutral about their outdoor view.
  - Classroom D: 50% are neutral, and 47% report no distraction from outdoor views, indicating minimal interference with focus. Only 3% find outdoor views distracting.
  - Classroom E: 57% are distracted by the outdoor views due to nearby street markets, garbage, and surrounding activity, which can significantly divert students’ attention from educational tasks. Additionally, 24% are neutral, and 19% report no distraction from outdoor views (see Fig. 10).

Students in these classrooms generally feel the lighting is favorable, indicating sufficient artificial and natural light for desk tasks and board visibility. However, the impact of outdoor views on concentration varies significantly across classrooms. This suggests that implementing strategies to reduce visual distractions, especially in classes C and E, may enhance students’ ability to focus.

These results clearly indicate the need for active intervention to improve the IEQ in schools, thereby fostering better and more comfortable learning environments. Effective Integrated policies that deal with noise, lighting, ventilation and thermal comfort all need attention. In particular, the following steps need to be planned.

#### Acoustic comfort (solely)

Students’ experiences with noise disruptions exhibit significant variability depending on the type of classroom, reflecting the unique layouts and environmental conditions inherent to each space:

- Classroom A: The nearest source of noise in this classroom is the playground, which constantly creates auditory disruptions due to its proximity to the classroom.
- Classroom B: The main source of disturbance in this classroom is the adjoining corridor, which is likely to be busy and full of activity.
- Classrooms C and E: Street noise is the most disturbing for students in these classrooms, and is usually associated with increased traffic on nearby streets.
- Classroom D: Most noise disruptions in this classroom originate from within the classroom, which is due to the high student density in the class.

With these differences, the effects of student density, classroom location, and the surroundings, as well as other factors, show their importance to the students’ acoustic experience (see Fig. 10). This figure illustrates the various types of noise present in the classroom and shows how these noises impact students’ learning and the quality of the indoor environment.

- Indoor Air Quality:
  - Schools can enhance indoor air quality by utilizing air purifiers in conjunction with improved ventilation systems.
  - Windows must be positioned strategically to optimize natural ventilation and air circulation, especially in areas with a high risk of external pollutants
- Thermal Comfort:
  - The use of thermal insulation and shading devices can help control classroom temperatures and significantly increase student comfort.
  - If these strategies are combined with natural ventilation techniques, these improvements can help stabilize temperatures during the school year.
- Visual Comfort:
  - Reducing glare from artificial lighting can be done by employing window designs that maximize natural light, while at the same time employing energy-efficient lighting systems.
  - This approach ensures providing enough light for desk and board tasks.
- Acoustic Comfort:
  - To reduce noise from streets, playgrounds, or corridors, sound-absorbing materials in walls, floors, and ceilings should be provided.
  - Placing students further from the noise sources allows for better focus and comfort which can be further enhanced by thoughtful classroom layouts and seating arrangements.

IEQ Education and Awareness: Educating and creating awareness regarding the staff and students in relation to the IEQ factors like noise, thermal comfort, air quality, and even visual comfort is important. Such awareness can be helpful in creating better ways of maintaining a conducive learning environment. Schools can decrease current discomforts and foster a more productive and healthier environment by taking these actions. All students and teachers will benefit from enhanced academic performance and wellbeing which directly results from this improved IEQ.

## Discussion

This research has shown that the effect of indoor environmental quality (IEQ) in the context of Egyptian classrooms is critically important, although understudied. The localized case studies performed in Nasr City, Cairo, serve as a starting point to analyze how certain behavioral environmental factors influence students’ academic engagement and satisfaction.

As evidenced by evaluating the students’ responses, indoor environmental quality (IEQ) significantly impacts students’ opinions and academic experiences in classrooms. While statistical data provide a quantitative overview of satisfaction levels, it is crucial to consider students’ individual experiences and various contextual factors, including classroom orientation, seasonal changes, and surrounding environmental conditions.

The study’s key findings reveal that students’ focus, comfort, and satisfaction are notably influenced by their seating location, window proximity, air quality, and thermal comfort. Previous studies have established that classroom positioning affects academic performance and student engagement levels^47^.

Interestingly, while students sitting near windows frequently reported higher energy levels and focus, those seated farther away noted feelings of sleepiness, which aligns with De Giuli’s research suggesting that proximity to windows enhances access to fresh air and natural light^10^. This finding is echoed in recent studies demonstrating that natural lighting can reduce fatigue and improve cognitive function among students^48^.

However, contrary to expectations, some students expressed discomfort even in well-ventilated classrooms, implying potential underlying issues such as nearby street markets that could compromise perceived air quality when windows are open. This complexity underscores previous findings by Son et al., which highlight that external environmental factors often overshadow internal air quality measures in urban settings^49^.

The challenges facing densely populated areas in arid climates provide important insights not only for Egypt but also for other developing countries seeking to enhance their educational environments. Moreover, the attention given to classrooms with natural ventilation features highlights a well-defined problem that can be addressed in Egypt. The study emphasizes the pressing need for innovative solutions tailored to local climate conditions as demonstrated by previous international research that has not yet been applied or adapted to the Egyptian context.

The findings regarding thermal comfort also revealed surprising patterns. Although many students reported discomfort when temperatures rose above optimal levels, many did not actively seek to adjust their environment, indicating a possible lack of awareness or agency regarding their comfort. Al Horr’s and Aguilar’s studies support this finding, showing that inadequate ventilation exacerbates discomfort^8,29^. Furthermore, a more recent research by Villarreal Arroyo et al. demonstrates similar findings as above by showing that students depend more on external environmental triggers instead of actively working on their thermal controls^50^. To address these concerns, simple and cost-effective interventions could include installing user-friendly operable windows or automatic ventilation systems that actively respond to indoor air quality changes and provide consistent airflow without relying solely on manual adjustment, which may not always occur.

Additionally, visual and acoustic comfort are imperative to the classroom experience. While ample natural lighting can enhance productivity, distracting outdoor views may decrease concentration. Visual distractions can impact learning results, which increases concern over classroom lighting design and efficient window placement in the classroom design^51^. Therefore, implementing window treatments that allow flexible control over light and view while minimizing distractions could significantly enhance learning environments.

Regarding noise, the study found that outdoor disturbances, such as traffic and playground sounds, detracted from student performance. This supports the findings from Magloire et al.'s study, stating that noise pollution is related to lower academic achievements in urban schools^52^. To combat this, incorporating sound-absorbing materials or technologies within classrooms can help mitigate disruptive noise levels and create a more conducive learning atmosphere.

This study concludes by emphasizing the urgent need for targeted interventions to enhance school IEQ. Suggestions include upgrading ventilation systems, optimizing artificial and natural lighting, and addressing acoustic issues through design and material choices. These evidence-based strategies aim to create healthier, more effective learning environments, ultimately supporting students’ academic achievement and well-being.

This study analyzes in depth how the findings relate to the existing literature on Indoor Environmental Quality (IEQ), this study strives to significantly impact the real-world challenges associated with indoor environmental quality in educational settings by focusing on actionable improvements.

## Conclusion

This study evaluates key factors of IEQ in elementary school classrooms with natural ventilation, emphasizing thermal comfort, noise level, visual comfort, and air quality. The study gathers students’ opinions about their learning environments by distributing questionnaires to five classrooms in five different schools—two private, two experimental language schools, and one public—with varying classroom sizes, proportions, and densities, highlighting similarities and variations in perspectives.

The results highlight the significant impact of indoor environmental quality on students’ performance during the school day. Students’ comfort and well-being were significantly affected by varying dust levels, scents, air quality, and satisfaction levels across schools. While some classrooms expressed great pleasure with the air quality, others pointed out factors that needed improvement, especially regarding ventilation or reducing exposure to pollutants outdoors. Furthermore, the correlation in some schools between students’ emotional states and seating arrangements suggests the benefits of thoughtful classroom design in enhancing student performance.

According to the replies, students are generally satisfied with their seating arrangements and that windows are frequently open for ventilation, except during January, a colder month. Nevertheless, there were differences in the students’ opinions regarding dust and indoor smells, their satisfaction with fresh air, and how energized or sleepy they felt. The impact of external factors on the perceived quality of the air is demonstrated by the fact that some schools reported continuous exposure to outdoor pollutants.

All schools received favorable visual comfort evaluations, including outdoor views and lighting quality. This suggests that most students are satisfied with the lighting on their desks and boards. This factor of IEQ correlates with studies indicating that better lighting is associated with less fatigue and better academic performance.

Students identified noise sources from playgrounds, classrooms, corridors, and streets, revealing noise as a broad challenge. Despite these noise disruptions, most students expressed satisfaction with their ability to hear the teacher, particularly when the teacher used a louder voice tone, highlighting that teacher clarity was maintained across various noise situations.

Assessments of thermal comfort varied with the season. Students generally reported feeling neutral in November, experiencing cold temperatures in January, and feeling warm in October and April. The students adapt to these changes in temperature by wearing clothes that are suitable for different temperatures. Their clothes in very cold temperatures have a 0.91 clo value, their clothes in cold temperatures have a 0.55 clo value, and their clothes in warm temperatures have a 0.49 clo value. The windows were usually closed in cold temperatures, sometimes open in cold temperatures, and always open in warm temperatures. These variances highlight the importance of natural ventilation in maintaining comfort throughout the school year.

Regarding satisfaction with students’ seating location, there was no discernible difference between the opinions of boys and girls. Still, it was observable the girls usually preferred to sit in the front rows and nearer to the windows, and the boys tended to prefer sitting in the back rows and far away from the windows.

The degree of satisfaction with indoor air quality did not differ by gender. This dissatisfaction most likely suggests that the ventilation patterns are not adequately addressing odor problems within and outside the classroom. The closer the students are to the windows, the more irritated they are from the glare on their desks and the less clearly they hear their teacher’s voice. But on the other hand, these students tend to be satisfied with the amount of fresh air and the airflow in their classroom.

Classroom proportions do not significantly impact students’ satisfaction with IEQ factors. Instead, external factors like ventilation patterns and noise sources significantly influence students’ comfort and satisfaction. The surroundings around the school also considerably affect the indoor environmental quality of its classrooms, as outdoor pollutants and distractions from the streets all affect and disturb the students. Also, student density, classroom location, and surrounding environments can all affect students’ satisfaction and performance.

This investigation highlights that the proportions of classrooms do not significantly affect students’ satisfaction with the educational indoor quality (IEQ) factors; instead, the focus should be shifted to external factors, such as ventilation design and noise sources. For further improvement of the IEQ in elementary schools, the following recommendations are presented:

1. Better Ventilation Systems: In regions that suffer from high outdoor pollution, Schools should install advanced air ventilation systems that bring in fresh air while regulating the room’s air quality.
2. Use of Acoustic Materials: Installing soundproof materials in the classroom will reduce the discomfort caused by external noise, as well as enhance comfort with the sound.
3. Lighting Improvements: Modern, glare-free lighting fixtures will enhance lighting comfort, further improving concentration for students.
4. Systematic Monitoring of the Surrounding Environment: The school requires an internal monitoring system to control the quality of the air, the thermal environment, and the level of noise, allowing for prompt and relevant actions to be taken.
5. Classroom Arrangement Recommendations: Implementing recommendations that position desks near windows and away from noise sources will effectively maximize student satisfaction and performance.

This study’s conclusion highlights the importance of improving indoor environmental quality (IEQ) in elementary schools to create a favorable learning environment. The results highlight that promoting well-being and academic achievement requires a comprehensive approach that addresses thermal, air quality, visual, and acoustic comfort. This study highlights the significance of elementary school multi-strategy approaches in enhancing Indoor Environmental Quality (IEQ) within the school. To enable students to be healthy and productive academically, thermal comfort, air quality, and visual and acoustic conditions must be maintained within reasonable limits. The research aims to address this gap by proposing a framework for further study and the design of interventions to optimize environmental quality in Egyptian schools, thereby contributing to the existing literature. The intervention aimed to enhance student comfort, health, and productivity in a practical manner.

### Implications

The results of this research conducted stress the importance of improving Indoor Environmental Quality (IEQ) in educational facilities. There are multiple groups for whom this activity is pertinent: researchers, practitioners, policymakers, users/occupants, and civil society are all relevant stakeholders.

1. **Researchers:** The provided results have some relevance. However, additional research is necessary, particularly regarding longitudinal studies that aim to determine the effects of changes in Internal Environmental Quality (IEQ) on students’ performance and health over time. Other studies also need to evaluate numerous IEQ intervention approaches in different educational environments and assess the impacts of variability resulting from classroom and room design, ventilation systems, and external environmental conditions ^8^.
2. **Practitioners:** The noted gaps can be addressed within a short timeframe by school administrators and teachers, as the study proposes, by modifying the learning environment. For example, improving ventilation systems, enhancing classroom layouts to improve visual and acoustic comfort, and even soundproofing external walls. Additionally, there is a need for teacher training that enables adequate responses to students’ IEQ dissatisfaction, aiming to change the default culture of teacher responsiveness ^53^.
3. **Policymakers:** The study covers relevant aspects that can help inform decisions about the distribution of resources and funding towards particular schools. For example, improvement initiatives should be integrated into policies that aim to transform educational environments, especially those that facilitate and foster education. New laws must be created to establish minimum standards or benchmarks for IEQ in schools, or to provide incentives for schools that implement and sustain processes and technologies that enhance indoor environments. Additionally, there must be a built-in systematic evaluation of the condition of schools with regard to their indoor environmental quality (IEQ) in the policies in place ^54^.
4. **Users:** The primary educational space users, mostly students and teachers, have the first obligation to raise these issues regarding better practices of IEQ. The concepts of Indoor Environmental Quality (IEQ) should be presented to them in a manner that enables them to articulate their needs effectively.
5. **Civil Society:** Local organizations have the opportunity to utilize the findings from this research to raise awareness about the connection between health and education. Together with schools, these groups can engage parents and community leaders to support the improvement of the learning conditions. Multi-organizational initiatives aimed at improving school indoor environmental quality (IEQ) can have a significant impact at the community level, promoting the movement towards creating supportive educational environments that lead to improved health and educational outcomes for students^55^.

### Limitations

Limitations on sample size, data collecting permissions, and the targeted age group were the main limitations of this study, which affected the findings’ broader significance. The sample was further restricted by the requirement for security clearance from the Central Agency for Public Mobilization and Statistics, which limited it to specific categories of classrooms in Nasr City. To promote generalizability, future studies should expand their demographic and geographical scope.

Due to Ministry of Education restrictions, the study did not use outside equipment or take in-class measurements, and pictures of school interiors were forbidden to preserve schools’ and students’ privacy. These limitations highlighted the need for future research with access to measurable IEQ data by restricting the scope of this study’s data collection to student-reported perspectives.

Despite a high response rate, the study’s limited sample size of 123 fifth-grade students provided for this study analysis. Still, it also limits the study’s relevance to a specific age range and educational stage. Future studies should include a broader range of middle and high school students to understand IEQ comprehensively. Teachers should also be involved in learning more about how they interpret IEQ.

The questionnaire used in this study, which was conducted in November and is based on the memorable thermal conditions for the three other different months, limited to one day each school in November, only briefly documented the students’ perceptions. A more thorough understanding of environmental influences throughout the school year might be obtained by conducting research throughout several months, which could capture seasonal and temporal changes in IEQ.

Future studies should also evaluate the effectiveness of treatments intended to improve IEQ in schools and fill in existing knowledge gaps in IEQ. Comparative studies of various improvement initiatives and long-term studies examining changes in IEQ over time might provide helpful information for creating improved and healthy learning environments in Egyptian elementary schools.

## Data Availability

The data that support the findings of this study are available on request from the corresponding author.
